# Blink-related arousal network surges are shaped by cortical vigilance states

**DOI:** 10.21203/rs.3.rs-4271439/v1

**Published:** 2024-05-10

**Authors:** Sukru Demiral, Christina Lildharrie, Esther Lin, Helene Benveniste, Nora Volkow

**Affiliations:** National Institute on Alcohol Abuse and Alcoholism, National Institutes of Health,; National Institute on Alcohol Abuse and Alcoholism, National Institutes of Health,; National Institute on Alcohol Abuse and Alcoholism, National Institutes of Health,; Yale School of Medicine; National Institute of Drug Abuse

**Keywords:** arousal, vigilance, eye-blinks, HCP, fMRI

## Abstract

The vigilance state and the excitability of cortical networks impose wide-range effects on brain dynamics that arousal surges could promptly modify. We previously reported an association between spontaneous eye-blinks and BOLD activation in the brain arousal ascending network (AAN) and in thalamic nuclei based on 3T MR resting state brain images. Here we aimed to replicate our analyses using 7T MR images in a larger cohort of participants collected from the Human Connectome Project (HCP), which also contained simultaneous eye-tracking recordings, and to assess the interaction between the blink-associated arousal surges and the vigilance states. For this purpose, we compared blink associated BOLD activity under a vigilant versus a drowsy state, a classification made based on the pupillary data obtained during the fMRI scans. We conducted two main analyses: i) Cross-correlation analysis between the BOLD signal and blink events (eye blink time-series were convolved with the canonical and also with the temporal derivative of the Hemodynamic Response Function, HRF) within preselected regions of interests (ROIs) (i.e., brainstem AAN, thalamic and cerebellar nuclei) together with an exploratory voxel-wise analyses to assess the whole-brain, and ii) blink-event analysis of the BOLD signals to reveal the signal changes onset to the blinks in the preselected ROIs. Consistent with our prior findings on 3T MRI, we showed significant positive cross correlations between BOLD peaks in brainstem and thalamic nuclei that preceded or were overlapping with blink moments and that sharply decreased post-blink. Whole brain analysis revealed blink-related activation that was strongest in cerebellum, insula, lateral geniculate nucleus (LGN) and visual cortex. Drowsiness impacted HRF BOLD (enhancing it), time-to-peak (delaying it) and post-blink BOLD activity (accentuating decreases). Responses in the drowsy state could be related to the differences in the excitability of cortical, subcortical and cerebellar tissue, such that cerebellar and thalamic regions involved in visual attention processing were more responsive for the vigilant state, but AAN ROIs, as well as cerebellar and thalamic ROIs connected to pre-motor, frontal, temporal and DMN regions were less responsive. Such qualitative and quantitative differences in the blink related BOLD signal changes could reflect delayed cortical processing and the ineffectiveness of arousal surges during states of drowsiness. Future studies that manipulate arousal are needed to corroborate a mechanistic interaction of arousal surges with vigilance states and cortical excitability.

## INTRODUCTION

Eye blinks are sensitive to arousal and attention (Maffei & Angrilli, 2019; Wood & Hassett, 1983), and are influenced by cognitive states (e.g. attentional allocation, transitions in information flow, etc.) (Ponder & Kennedy, 1927; Stern et al., 1984) and amenable to conscious modification. For instance, the state of consciousness of patients with persistent vegetative and minimally conscious states could be inferred by the posterior delta and alpha EEG frequency oscillations observed at blink moments (Bonfiglio et al., 2013; Bonfiglio et al., 2014).

Complementary findings emerge from pre-clinical studies documenting that blinking was associated with increases in arousal and with resetting of neural and vascular dynamics (Turner et al., 2022). The neuronal pathways that underlie blinking and their associations with the ascending arousal network (AAN) are critical to arousal (Singh et al., 2022). The AAN is comprised of various brainstem nuclei including serotonergic (dorsal, DR; and medial raphe, MR), noradrenergic (locus coeruleus, LC), dopaminergic (substantia nigra, SN; and ventral tegmental area, VTA) and cholinergic (pontis oralis, PO; pedunculopontine, PPN) nuclei that project to thalamus, striatum and cortex (Edlow et al., 2012). AAN nuclei through their extended connections can influence broad cortical areas through which they modulate arousal levels (Salhi et al., 2023). While the relationship between blinks, blink rates and the dopamine system has been widely investigated (Taylor et al., 1999) including clinical studies implicating D1 and D2 striatal receptors in blink rates (Demiral et al., 2022), the role of other AAN nuclei to blink events is limited. In humans, we recently showed that in AAN and thalamic nuclei and also in cerebellar nuclei peak BOLD activity correlated with blink moments, which were then followed by broad anti-correlations, including sensory-motor regions, insula, amygdala, parahippocamal regions, and temporal cortex, as BOLD activity decreased post blink (Demiral et al., 2023). In the same study we also showed that during performance in a gambling task, the button presses and blinks were highly synchronized for rewarding trials, revealing a link between blinks and arousal surges with attention and motivation.

Here we aim to replicate our initial results linking eye blinks to the arousal system, which were obtained with a 3 Tesla MRI, on a larger data set obtained with 7 Tesla MRI, which provides with better temporal and spatial resolution and to extend the analyses to the association with vigilance as estimated based on pupillary data. For this purpose we used the HCP dataset of participants scanned with a 7T MRI with eye-tracking and measured the lagged cross-correlations between eye-blink related fluctuations (canonical and temporal derivative of Hemodynamic Response Function, HRF, convolved blink time series, see **Supplementary Figs. 1 and 2**) and BOLD signal fluctuations in preselected regions of interest (ROI) extracted from AAN (Edlow et al., 2012) and thalamic nuclei (Behrens et al., 2003) from resting state fMRI images using temporal lags (TRs) of − 10s to + 10s. We also extracted ROI in cerebellar nuclei (Buckner et al., 2011), since they receive direct projections from brainstem (Singh et al., 2022) and are implicated in blinking (Dimitrova et al., 2002).

Our first hypothesis was that eye blinks would be in temporal correspondence with activation in AAN, thalamic and cerebellar nuclei such that their activity would peak in temporal association with eye blinks coincident with or preceding them.

Our second hypothesis was that the cortical vigilance state would influence arousal surges and blink related BOLD signals such that i) drowsiness would impose higher amplitude fluctuations and higher variance in the blink related BOLD response due to changes in cortical excitability (see below), and ii) delay in blink related neural response initiated by arousal surges (delayed time difference between blink associated neural activity and the blink itself) due to delays in neural communication.

In our paper we refer to ‘arousal’ as the physiological state driven by the ascending brainstem and thalamic arousal nuclei required to keep the organism *attentive* and *conscious*, and refer to ‘drowsiness’ (somnolence) as the cortical transitional state between wakefulness and sleep, observed under non-engaging or routine environments or under conditions of sleep deficits. In particular, while conducting resting state MRI scans, participants lay supine under dimmed lights, which can make some individuals feel drowsy and disengaged with a concomitant shutting down of their external senses, a phenomenon known as micro-sleep (Poudel et al., 2014; Soon et al., 2021). These episodes can have partially overlapping features and behavioral presentation with fatigue or tiredness (i.e., sleepiness and fatigue often coexist as a consequence of sleep deprivation) (Shen et al., 2006) or merely emerge due to losing interest in and disengage from the setup in the scanner environment (since there is nothing to do in the resting state), or due to inconsistent activity in systems that regulate sleep; for instance orexin neurons contribute to the maintenance of arousal by inhibiting sleep-promoting neurons in the ventrolateral preoptic nucleus (De Luca et al., 2022). Both sleep deprivation and drowsiness interfere with performance (Makeig et al., 2000; Van Dongen et al., 2003) increasing missed or delaying responses to external task stimuli (i.e., psychomotor vigilance task, PVT).

Drowsiness and arousal can interact with each other, an interaction that has been most commonly described in the sleep literature, where arousal related activity has been found to influence sleep stage changes (Horner et al., 1997; Jones, 2020). Similarly, in the MRI environment, arousal surges can emerge to help the person stay awake and attentive and overcome drowsiness and sleep drive.

Cortical excitability as a function of vigilance has been assessed with Transcranial Magnetic Stimulation (TMS). Specifically TMS evoked EEG potentials (TEP) has been used to measure the propagation of activity from the site of stimulation, thereby providing information on the excitability of brain networks (Sulcova et al., 2022). For example, a recent study showed that prolonged wakefulness and sleep deprivation related drowsiness increased the immediate (< 30ms) TEP EEG response (both amplitude, theta power, and slope to peak-time) to TMS, which was rebalanced after one night of sleep (Huber et al., 2013). Another study suggested that sleep deprivation upscales cortical excitability due to enhanced glutamate-related cortical facilitation and decreases and/or reverses GABAergic cortical inhibition (Salehinejad et al., 2022), probably by engaging homeostatic factors that modulate these neurotransmitter systems (Del Cid-Pellitero et al., 2017). (However, see (Mroczek et al., 2024) for a comparison and contrast of the effects of different TMS techniques on a set of cortical excitability measures).

In this context, we hypothesized that blinking and its temporal proximity to neural activation in the arousal systems would be impacted by drowsiness, such that the tonic cortical responsiveness during states of decreased vigilance would lead to slower responses to the transient fluctuations of arousal surges. Indeed, sleep deprivation and states of drowsiness have been shown to delay responses to task stimuli (i.e., in PVT) (Makeig et al., 2000; Van Dongen et al., 2003). Considering the reported increased neuronal excitability to TMS in states of drowsiness, we also hypothesized enhanced blink-associated BOLD reactivity. Thus, we expected that blink-related neural excitability would increase, and responses would be delayed under drowsiness.

To determine the effects of vigilance on BOLD-blink associations we relied on the pupillary data to estimate the percentage of time the eyes were open during an fMRI run; defining ‘Vigilant’ when the eyes were open > 90% and ‘drowsy’ when the eyes were closed between 10%–75% of the time. We found that cross-correlations were strongest preceding or coincident with the blink within AAN and thalamic nuclei (also in cerebellum) replicating our prior findings, and that vigilance influenced these associations, affecting their amplitude and timing. Brain-wide analyses also identified strong positive cross-correlations in insula, lateral geniculate nucleus (LGN) and visual cortex, and wide cortical anti-correlations post blink that were driven by the state of drowsiness in sensory-motor and temporal cortices. Complementing these analyses, using the temporal derivative of the HRF, we show that time-to-peak of the blink-related BOLD activity occurred earlier in the drowsy than in the vigilant state as the HRF rise from the neural activity was delayed. The event-based analysis also showed that blinks were delayed after the initiation of arousal surges in the drowsy compared to the vigilant state. Our results revealed vigilance-dependent blink timing coincident with arousal and cortical networks in the brain.

## Materials and Methods

### Data

We used a subset of the Human Connectome Project (HCP) dataset (Van Essen et al., 2013) acquired on the 7T system made publicly available as part of the 1200 Data Release (March 2018). This data subset consists of 723 resting-state scans (15 mins each) acquired on a group of 184 subjects (each subject had up to four resting state runs distributed in two days). Concurrent eye pupil traces are available as part of the data release. Relatively short TR (1s) enabled us to obtain better temporal resolution than in our prior analyses (Demiral et al., 2023). Scanning parameters for this data are TR = 1s, TE = 22.2ms, FA = 45°, voxel resolution = 1.6 × 1.6 × 1.6mm^3^, Multiband Factor = 5, GRAPPA = 2 phase direction: AP or PA. Additional details can be found on the Reference Manual for the 1200 HCP Release available online at https://www.humanconnectome.org/storage/app/media/documentation/s1200/HCP_S1200_Release_Reference_Manual.pdf.

Participants were positioned supine in the MR scanner and during the resting state scan stared at a cross on the center of the screen together with eye-tracking. In total, we found that 177 subjects had four runs of resting state fMRI scans, two had three runs, four had two and one participant had only one run in the database. Among these runs some lacked eye tracking data leading to 131 participants with four runs, 14 participants with 3 runs, one with two runs and two participants with one run of combined resting state and eye tracking data available (leading to 570 runs across 148 participants). Of these, 10 runs had to be excluded due to problems in the eye-tracking file either not being recorded properly or showing problems in opening/importing files into the computer software. Final set comprised 560 runs across 148 participants. Further vigilance categorization of the eye-tracking data is explained below.

### fMRI pre-processing

HCP data is initially minimally pre-processed (Glasser et al., 2013), which includes distortion correction, motion correction, bias field correction and spatial normalization to the MNI template space. HCP fMRI data was further processed by the same group of researchers with i) applying weak high-pass temporal filtering (> 2000s FWHM) achieving slow drift removal, ii) running Independent Component Analysis (ICA) based correction method (MELODIC ICA, where artefact components were identified - the procedure labelled as ICA-FIX), and iii) regressing out artefact and motion-related time courses (Smith et al., 2013). Thus, we used the final cleaned datasets labelled as “rfMRI_RESTx_7T_PA_hp2000_clean.nii.gz”. Here the ‘RESTx’ specifies the order of the resting state scan of the participant (i.e., REST1, REST2 etc.), and ‘AP’ specifies either AP or PA phase direction, and ‘hp2000’ is the high-pass filtering value (T = 2000s, f = 1/2000s). In addition, since the first few volumes of a functional acquisition may contain large signal changes, which stabilize as the tissues reach steady state later in time, we discarded the initial 10 volumes (thus we used 890s of both the eye-tracking and fMRI data). In addition, low-frequency scanner drifts and linear trends were filtered with band-pass filtering (0.01–0.1 *Hz*). This data was then used for the preselected ROI-based analyses. For the whole-brain analysis and image reports, we additionally applied spatial smoothing (*FWHM = 5mm*). The resulting signal was then used for cross-correlation analysis with the blink regressors extracted as described below. We also calculated framewise displacement (FD) as motion estimates, which did not differ between vigilance states (see **Supplementary Fig. 3** for FD distributions of runs per vigilance state).

### Eye-tracking pre-processing

Eye-tracking was conducted with the Eye Link 1000 system with 1kHz sampling rate (some runs were 500Hz) from the Right eye (note that a few subjects had Left eye recording). We conducted the following pre-processing steps: i) Extracted all runs with usable continuous eye-tracking data, ii) runs with 500Hz sampling rate were up-sampled to 1kHz, iii) time period between 40–400ms of pupil loss was marked as eye-blinks (blink onset moment was the starting time of the pupil loss), iv) missing data points due to eye-blinks were linearly interpolated with the neighboring points, v) pupil size time series were finally down sampled to 100Hz and smoothed with a gaussian kernel (FWHM of 20 sampling points; 200ms). (See (Gonzalez-Castillo et al., 2022) for similar eye-tracking pre-processing steps.)

### Vigilance classification

Each resting scan/run was initially classified into one of four states: a) vigilant (when the pupil was not detected for less than 10% of the scan time in a run); b) drowsy (10%–40% pupil loss in a run); c) very drowsy (40%–75% pupil loss in a run); and d) discarded (more than 75% pupil loss). (See **Supplementary Figs. 3 and 4** for histogram of runs according to vigilance classification and plots showing the state of pupil change during the scan.) Of the total of 560 runs from the 7T HCP dataset 291 were classified as vigilant runs (52% of the total runs across 111 subjects), 111 as drowsy runs (19.8% of the total runs across 75 subjects), 85 as very drowsy runs (15.2% of the total runs across 59 subjects) and 73 were discarded (13% of the total runs across 41 subjects). 58 subjects had both vigilant and drowsy runs available; 32 subjects had both vigilant and very drowsy runs, and 68 subjects had vigilant and any type of drowsy runs. We excluded one run with less than a total of 15 blinks per run (~ 1 blink per minute) to obtain more reliable blink measure (i.e., blink duration, blink rate etc.) After exclusion of the ‘discarded’ runs, final total number of runs were 486, distributed across 141 participants. We combined drowsy and very drowsy runs, which we labelled as “Drowsy” and used to compare “Drowsy” state against “Vigilant” state.

### Blink behavior across vigilance states (drowsy and vigilant)

We report inter-blink interval (IBI) and blink duration distributions as histograms using all the blink events per vigilance status. Additionally, we present boxplots of blink rate (blinks per minute then the pupil was available, not the whole run time), IBI, and blink duration, and ran one-way ANOVAs to compare differences between vigilance states using mean values per run. In this analysis we excluded outliers (Median±1.5*IQR) (**Supplementary Fig. 5** for Inter-blink interval (IBI) and **Supplementary Fig. 6** blink duration histograms and box-plots comparing vigilance states on eye blink measures)

### Blink BOLD cross-correlation analysis

For each scan series, blink onset time was used as a unit stick function to construct a time series, which was then separately convolved with the canonical HRF (2-gamma function) and the temporal derivative of the HRF, and normalized (*z*-scored) across time (See **Supplementary Fig. 2** for an example of canonical HRF time series convolution). These time series were then used for cross-correlation analysis with (i) voxel-wise averaged temporal BOLD activity in each preselected ROI, and (ii) activity of each voxel in the whole brain analyses, in each temporal TR (1s) lag starting from − 10s up to + 10 seconds, and the correlations were transformed to Fisher’s z-values. Negative values indicated BOLD preceding blink, and positive values indicate BOLD following blink. In the group-level statistical analysis in SPM12, we used these z-values per run divided into two categorical levels of vigilance states (vigilant or drowsy) together with blink rate per run as covariate. Final statistical reports are presented as t-values (i.e., vigilant, drowsy and vigilant – drowsy difference).

### ROI analyses in AAN and thalamic nuclei, and cerebellum

In the AAN, we computed correlation activity for 10 ROIs extracted from catecholaminergic and cholinergic brainstem nuclei from the AAN atlas (Bianciardi et al., 2015; Edlow et al., 2012)(https://www.nmr.mgh.harvard.edu/resources/aan-atlas); using a 1-mm, MNI152 template, with the addition of left and right SN pars compacta (SMpc) regions from the Automated Anatomical Labelling Atlas (https://www.oxcns.org/aal3.html) (Rolls et al., 2020). The AAN nuclei corresponded to the serotonergic dorsal (DR) and median raphe (MR), the noradrenergic LC, the dopaminergic VTA and SNpc, and the cholinergic nuclei PO and pedunculopontine nucleus (PPN). Additionally, we also extracted ROI in periaqueductal gray (PAG), mid-brain reticular formation (MRF), parabrachial complex (PBC), similar to our previous publication (Demiral et al., 2023).

In the thalamus, we extracted seven ROIs from the atlas defined by Behrens et al. (Behrens et al., 2003; Demiral et al., 2023; Demiral et al., 2019), which is based on white matter connectivity probabilities with major cortical areas (i.e. motor, premotor, frontal, occipital, temporal, parietal and sensory cortices).

In the cerebellum, we extracted seven ROIs from the atlas defined by Buckner et al. (Buckner et al., 2011), which show resting-state functional connectivity probabilities with major cortical networks (i.e. visual, sensory-motor, dorsal attention, ventral attention, orbito-frontal, fronto-parietal control, default mode (DMN) networks.

### Statistical analysis of the ROI correlations

Since our analysis showed that pre-blink maximum and post-blink minimum correlations in the canonical HRF cross-correlation analysis changed due to the vigilance state (see below), we quantified the effect of vigilance on the correlation measures with regression analyses using continuous vigilance values (i.e., eye-closure) and the max/min correlation lag-times and amplitudes. Before running these analyses, we pooled all the correlation values (total of 21 values ranging from − 10s to + 10s) per run and per ROI, and excluded the outliers (i.e., limits median±1.5*IQR (inter-quartile range). For ROI analysis, we report the BOLD—eye-measure lagged-correlations as line plots. In this analysis, we used spatially unsmoothed BOLD values.

### Statistical analysis for the whole-brain

For the whole-brain voxel-wise brain activations, cluster correction (k > 10) with a t-value threshold of 10 applied (p < 5E-8) for the main effects (i.e., vigilant and drowsy), and t-value threshold of 5 applied for the difference (vigilant-drowsy). For the smaller brainstem regions, which are generally statistically weaker due to lower SNR and smaller cluster sizes), when we present brain maps of these regions, we used t-value threshold of 3. We refer to “pre-blink” when BOLD activation precedes blink (left-side of the x-axis), and “post blink” when BOLD signal occurred after the blink (right-side of the x-axis). Note that for the whole brain analysis we used smoothed (FWHM = 5mm) images.

### Blink-event BOLD signal analyses

In the final analysis, we selected blink epochs (BOLD signals within − 8s before blinks to + 15s after blinks) with a certain criterion to be able to detect real-time blink-event based BOLD signal fluctuations in the ROIs. Because blinks happen in temporal proximity to each other (see Supplementary Fig. 5 for IBI), it is difficult to obtain large numbers of isolated blink epochs for a good enough event signal. Thus, while imperfect, we applied the following procedure for depicting blink-event BOLD signal: We accepted any blink event (B0) only if i) it followed the previous blink event (B−1) by at least 4s to reduce noise over the baseline interval which was selected as between [−4s to −2s], and ii) if the next blink event (B + 1) was within the 2s interval from B0 (close-proximity blink cluster), but iii) rejected the blink event B0 if B + 1 was within 2s-8s away from B0 to reduce the noise in the post-blink interval, and finally iv) included B0 if B + 1 was at least 8s away from B0. We refer to this approach as “blink clustering’ since blinks close to each other (0s – 2s) were treated as if they were a single event (with a compromise of time resolution of 2s), and the following blink (B + 1) was never selected as a new epoch again later since it fell in a proximity to its previous neighbor (B0). This approach ensured that we did not inflate and re-introduce blink events, and obtain increased SNR, but on the other hand, it did not fully control for the potential effects of the upcoming blink events (i.e., B + 2 etc.) on the B0 epoch BOLD signal. We used a total of 15717 blink epochs for the Vigilant state, and 7731 blink epochs for the drowsy state.

## RESULTS

### Eye-behavior

Plotting IBI and blink duration histograms revealed that IBI had higher kurtosis and skewness values (300 and 12) in the Drowsy compared to the Vigilant state (61 and 5.7). In the Vigilant state, a single peak emerged around .8s whereas in the Drowsy state two peaks emerged around .4s and .8s. Right tail of the distributions became narrower for the longer IBIs (**Supplementary Fig. 5**). Blink duration in the Drowsy state was somewhat platykurtic (lower kurtosis; kurtosis = 2.22, skewness = .08) than in the Vigilant state, which showed leptokurtic distribution (higher kurtosis; kurtosis = 2.87, skewness = .41). In general, peak durations peaked around 150–200ms (**Supplementary Fig. 5**). Randomization based distribution of 10,000 values (random selection of 10,000 values and calculating the differences in the kurtosis and skewness) for both IBI and blink durations showed that the Vigilant-Drowsy differences were significant (p < .001, see **Supplementary Figs. 7 and 8**).

One-way ANOVA analyses showed that blink rate (blinks per minute eyes open) (F(1,470) = 49.9, p < .001) and blink duration (F(1,485) = 6.1., p < .05) were significantly higher in the Drowsy compared to the Vigilant sate. IBI was lower in the Drowsy than the vigilant state (F(1,426) = 47.6, p < .001).

### Cross-correlation analysis: Canonical HRF analysis

#### ROI analysis

**Figure 1:**
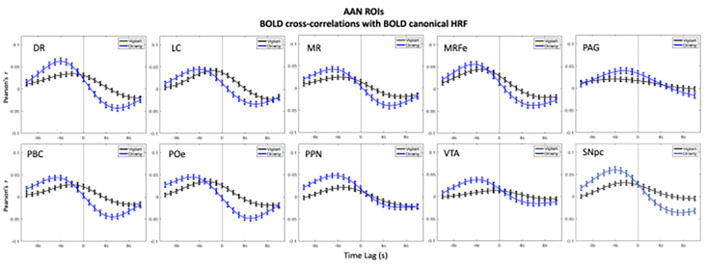
BOLD - Blink (canonical HRF convolved with blink time series) cross-correlations in AAN ROIs. Blink time series are convolved with canonical HRF and correlated with the AAN ROI BOLD time series. Negative values in the x-axis indicate BOLD preceding the blink time series and positive values indicate BOLD following the blink time series, and zero value in x-axis is where 0-lag correlation is measured. Blue lines show the Drowsy runs, black lines show the Vigilant runs. Abbreviations correspond to dorsal raphe (DR), locus coeruleus (LC), median raphe (MR), midbrain reticular formation (MRF), periaqueductal grey (PAG), parabrachial complex (PBC), pontis oralis (PO), pedunculopontine nucleus (PPN), ventral tegmental area (VTA) and substantia nigra pars compacta (SNpc).

**Figure 2:**
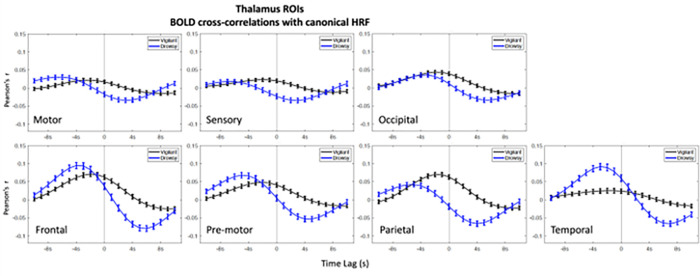
BOLD - Blink (canonical HRF convolved with blink time series) cross-correlations in Thalamus ROIs. Blink time series are convolved with canonical HRF and correlated with the Thalamus ROI BOLD time series. Negative values in the x-axis indicate BOLD preceding the blink time series and positive values indicate BOLD following the blink time series, and zero value in x-axis is where 0-lag correlation is measured. Blue lines show Drowsy runs, black lines show Vigilant runs.

**Figure 3:**
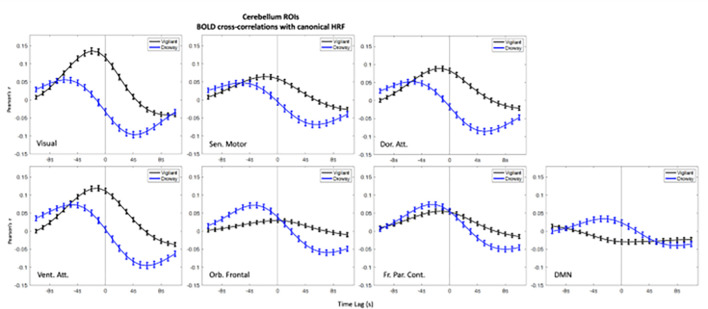
BOLD – Blink (canonical HRF convolved with blink time series) cross-correlations in Cerebellar ROIs. Blink time series are convolved with canonical HRF and correlated with the Cerebellum ROIs. Blink time series are convolved with canonical HRF and correlated with the Cerebellum ROI BOLD time series. Negative values in the x-axis indicate BOLD preceding the blink and positive values indicate BOLD following the blink time series, and zero value in x-axis is where 0-lag correlation is measured. Blue lines show the Drowsy runs, black lines show the Vigilant runs.

In the ROIs analyses higher positive correlations emerged in the negative lags (BOLD preceding blinks) and higher negative (anti-correlations) emerged in the positive lags (BOLD following blinks) (Figs. 1–3). This bi-phasic pattern was observed for most ROIs. The Drowsy state exposed largest maximal and minimal values with earlier peaks for almost all AAN and thalamic nuclei except in the thalamic Parietal ROI. In contrast for the Cerebellum greater peaks were observed in the Vigilant than the Drowsy state in Visual, Sensory Motor, Dorsal and Ventral Attention ROIs, though greater minimal values were observed for the Drowsy state in all ROIs.

### Pre-blink maximum and post-blink minimum and vigilance regression analysis

Using the values from the curves depicted in Figs. 1, 2 and 3, we extracted pre-blink maximum and post-blink minimum values at corresponding lags and computed the correlations between vigilance level (continuous eye closure ratio in y-axis, higher values longer closures) and the correlation values and time lags (x-axis, higher values more positive lags) obtained from the cross—correlation analysis reported across runs (Regression plots are presented in **Supplementary Figs. 9–11**).

### AAN

The correlations with maximal pre-blink amplitudes were positive and significant for VTA (r = .15, p < .005*; ‘*’multiple comparison corrected alpha = .05/10 = .005) and negative for the minimum post-blink amplitude for DR (r=−.19, p < .001*), LC (r=−.16, p < .005*), MR (r=−.14, p < .005*), MRF (r=−.15, p < .005*), PBC (r=−.19, p < .001*), PO (r=−.18, p < .001*), and SNpc (r=−.19, p < .001*). (See **Supplementary Fig. 9**).

Maximum pre-blink latency showed positive correlation only in PO (r=−.13, p = .005*) and for post minimum post blink latency none were significant. We also repeated the amplitude and lag analyses using the ‘peak’ values and report results under **Supplementary Report Part I, Supplementary Fig. 12**.

#### Summary

The amplitudes of positive-lag minimum values had a strong association with vigilance while the amplitudes of negative-lag maximum values showed weaker associations with vigilance.

### Thalamus

Maximal pre-blink amplitudes were positively correlated in Temporal ROI (r = .22, p < .001*; * indicates multiple comparison corrected alpha = .05/7 = .007) and negatively correlated in Parietal ROI (r=−.17, p < .001*). Minimal post-blink peak amplitude showed negative correlations for Sensory (r=−.17, p < .005*), Occipital (r=−.014, p < .005*), Frontal (r=−.21, p < .001*), Pre-motor (r=−.21, p < .001*), Parietal (r=−.3, p < .001*), and Temporal ROI (r=−.17, p < .005*). **Supplementary Fig. 10** shows the regression plots for pre-blink max and post-blink min amplitudes (x-axis) with drowsiness (y-axis) in Occipital, Pre-motor and Temporal thalamic ROIs.

Maximal pre-blink peak latency showed negative correlations (higher the drowsiness earlier the peak) for Motor (r=−.2, p < .001*), Sensory (r=− .15, p < .005*), Pre-motor (r=−.13, p = .005*), Parietal (r=−.29, p < .001*) ROIs. Minimal post-blink latency were negatively correlated (higher the drowsiness earlier the minimum correlations – closer to the blink) for Motor (r=−.19, p < .005*), Sensory (r=−.15, p < .005*), Pre-motor (r=−.15, p < .005*) and Parietal ROIs (r=−.3, p < .001*). Results for the amplitude and lag analyses using the ‘peak’ values are reported in **Supplementary Report Part I, Supplementary Fig. 13**.

#### Summary

The Temporal and Parietal ROIs showed significant pre-blink and post-blink amplitude changes respectively, due to vigilance. Post-blink vigilance effects were also observed for Sensory, Pre-motor, and Motor ROIs (See **Supplementary Fig. 10**). In the lag analysis, the Temporal ROI was distinct in that max/min latency showed positive correlations in pre- and post-blink windows indicating that the rise and fall in the Vigilant state emerged earlier than in the Drowsy state, opposite to the Motor and Parietal ROIs.

### Cerebellum

Contrary to the patterns in AAN and thalamus, most pre-blink max values were ‘negatively’ correlated for visual (r=−.38, p < .001*, ‘*’ indicates multiple comparison corrected alpha = .05/7 = .007)), dorsal attention (r=−.21, p < .001*), and ventral attention. (r=−.21, p < .001*).

For min post-blink amplitudes; *negative* correlations were found for Visual (r=−.27, p < .001*), Sensory-motor (r=−.029, p < .001*), Dorsal attention (r=−.33, p < .001*), Ventral attention (r=−.33, p < .001*), Orbito-frontal (r=−.26, p < .001*) and Fronto-parietal control (r=.−2, p < .001*) ROIs. ). **Supplementary Fig. 11** shows the regression analysis plots for pre-blink max and post-blink min amplitudes (x-axis) with drowsiness (y-axis) for Visual, Ventral attention and DMN ROIs.

For max pre-blink latency, negative correlations (higher the drowsiness earlier the max) were found for Visual (r=−.35, p < .001*), Sensory-motor (r=−.19, p < .001*), Dorsal attention (r=−.3, p < .001*), Ventral attention ROIs (r=−.29, p < .001*), but positive correlation for DMN ROI (r = .16, p = .001*).

Post-blink max lag times were also negatively correlated (higher the drowsiness earlier the min) for Visual (r=−.32, p < .001*), Sensory motor (r=−.16, p = .001*), Dorsal attention (r=−.22, p < .001*), Ventral attention ROI (r=−.26, p < .001*). The amplitude and lag analyses using the ‘peak’ values is reported in **Supplementary Report Part I, Supplementary Fig. 14**.

#### Summary

The amplitude and lag correlations in cerebellum for the pre blink maximum were opposite to those in ANN and thalamic nuclei such that the higher the drowsiness the less positive the pre-blink. Max/min correlations in the Drowsy state emerged earlier in the pre-blink and post-blink windows compared to Vigilant in most ROIs except for DMN ROI.

### Whole-brain analysis (Canonical HRF cross-correlation analysis)

**Figure 4:**
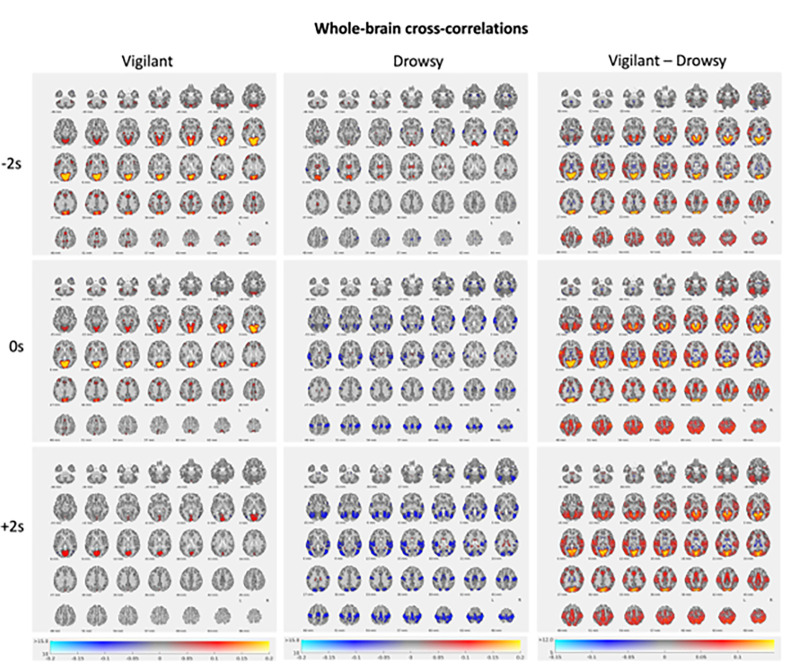
BOLD-blink (convolved with the canonical HRF) cross-correlations across the brain. Blink cross-correlations across the brain in time lags of −2s, 0s, and 2s where negative values indicate BOLD preceding and positive values indicate BOLD following the blink. Blink time series are convolved with canonical HRF. Correlation values (r) are shown in the x-axis of the color bar (from −.2 to .2) with t>10 for the main effects and (from −.15 to .15) with t>5 for difference.

Here we report lagged cross-correlations between canonical HRF convolved blink time series and BOLD signal for whole brain voxels. Statistical results for t-value threshold of 10 are presented for − 2s, 0s, and + 2s time lags in axial view in Fig. 4. Full results are presented in Table 1 for 0s-lag. We observed that pre-blink correlations where stronger for the Vigilant state in visual cortex, LGN, cerebellum, anterior insula, and ACC compared to the Drowsy state whereas the thalamus was stronger for the Drowsy state (Fig. 4 for contrasts). During the 0s-lag for the Vigilant state the positive correlations observed in −2s lag persisted whereas for the Drowsy state negative correlations emerged in frontal and temporal cortices including fusiform cortex and amygdala. The positive correlation in the thalamus observed at −2s lag for the Drowsy state persisted but was much more localized.. For the + 2s lag the positive correlations in the Vigilant state only persisted for the visual cortex (but smaller), whereas for the Drowsy state the broad anti-correlations in the sensory-motor areas and temporal lobes further expanded.

Table 1 below summarizes the significant clusters thresholded for t > 10 and minimum 10mm^3^ with positive correlations shown in bold and negative ones in regular font.

### Temporal derivative of HRF

**Figure 5:**
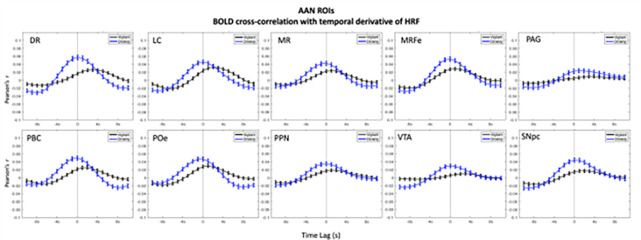
Blink-BOLD (temporal derivative of HRF convolved with blink time series) cross-correlations in AAN ROIs. Blink time series are convolved with temporal derivative of HRF and correlated with the AAN ROIs BOLD time series. Negative values in the x-axis indicate BOLD preceding the blink and positive values indicate BOLD following the blink, and 0 value in x-axis is where 0-lag correlation is measured. Blue lines show the drowsy runs, black lines show the vigilant runs. Abbreviations correspond to dorsal raphe (DR), locus coeruleus (LC), median raphe (MR), midbrain reticular formation (MRF), periaqueductal grey (PAG), parabrachial complex (PBC), pontis oralis (PO), pedunculopontine nucleus (PPN), ventral tegmental area (VTA) and substantia nigra pars compacta (SNpc).

**Figure 6:**
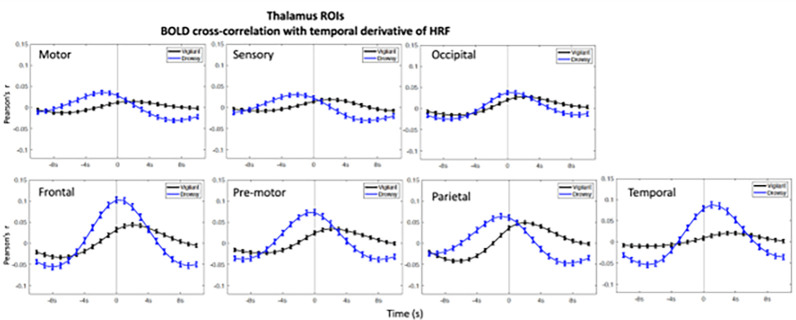
Blink-BOLD (temporal derivative of HRF convolved with blink time series) cross-correlations in Thalamus ROIs. Blink time series are convolved with temporal derivative of HRF and correlated with the Thalamus ROIs BOLD time series. Negative values in the x-axis indicate BOLD preceding the blink and positive values indicate BOLD following the blink, and 0 value in x-axis is where 0-lag correlation is measured. Blue lines show drowsy runs, black lines show vigilant runs.

**Figure 7:**
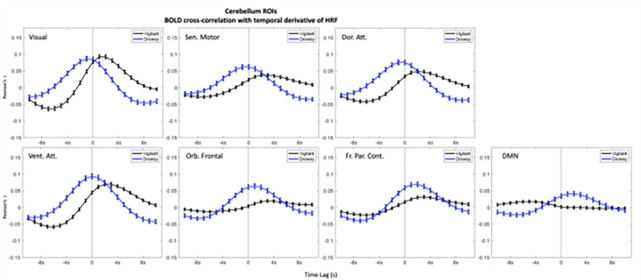
Blink-BOLD (temporal derivative of HRF convolved with blink time series) cross-correlations in Cerebellar ROIs. Blink time series are convolved with temporal derivative of HRF and correlated with the Cerebellum ROIs BOLD time series. Negative values in the x-axis indicate BOLD preceding the blink and positive values indicate BOLD following the blink, and 0 value in x-axis is where 0-lag correlation is measured. Blue lines show the drowsy runs, black lines show the vigilant runs, and red lines show all runs.

The canonical HRF convolution assumes standard time-to-peak values defined by the HRF and does not consider the brain region nor the state of vigilance. However, the time-to-peak and the shape of the HRF BOLD response might differ between brain regions and for different subjects (Handwerker et al., 2004; Rangaprakash et al., 2018) with substantial HRF shape variability (Chen et al., 2023). Vigilance could also impact HRF responses due to excitability changes. Using the temporal derivative of HRF allowed us to observe temporal shifts in time-to-peak and phasic increases/decreases with greater precision. This approach revealed that the highest correlations in AAN and thalamic nuclei and cerebellar regions emerged close to the blink and were higher for the Drowsy than the Vigilant state, for which the maximal correlations were shifted forward in time compared to the Drowsy state (Figures 5–7). Whole-brain analysis for the temporal derivative of HRF also revealed that time -to-peak times differed between vigilance states where the pre-blink time points had stronger correlations for the Drowsy state indicating that HRF initiation and peaking emerged earlier than in the Vigilant state (**Supplementary Report Part II**, **Supplementary Figure 15**).

The regression analysis between max/min correlation around blinks for the temporal derivative of HRF confirmed that as drowsiness increased higher correlations were observed at earlier time lags (**Supplementary Report Part II**). The shorter time-to-peak delays in HRF in the Drowsy state are consistent with delayed blinking relative to the ‘initiation of the neural activity’.

### Blink-event BOLD signal changes

**Figure 8:**
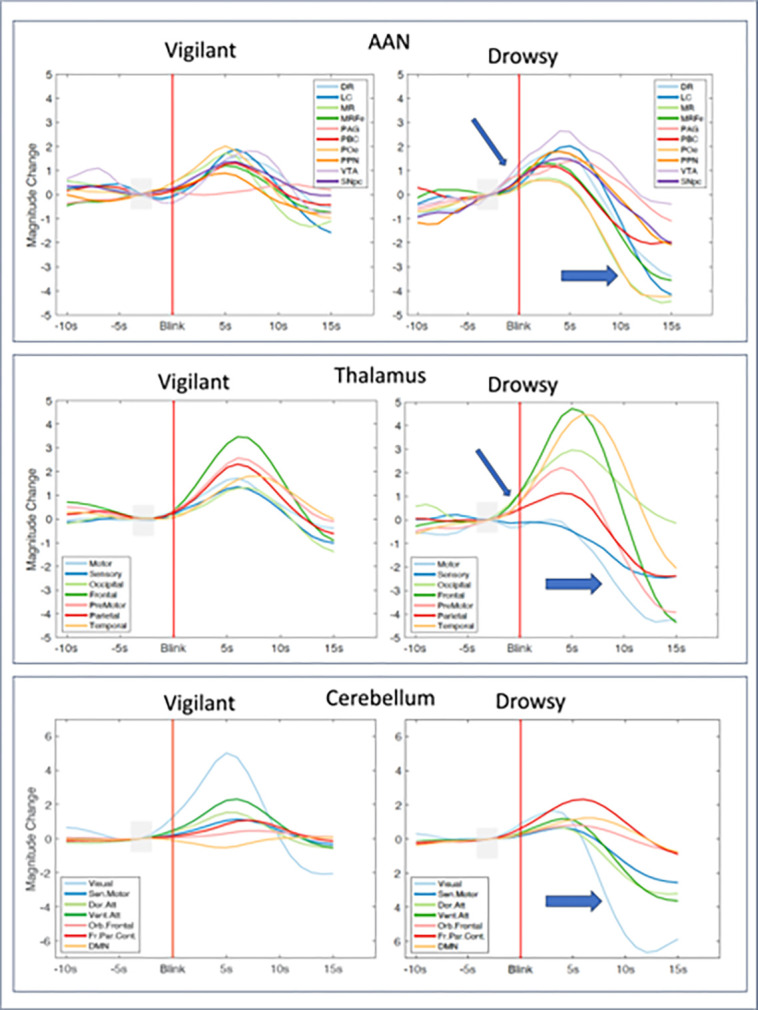
Blink-event based BOLD signal changes shown together. AAN, Thalamus, and Cerebellum ROIs grouped together in Vigilant and Drowsy states. The initiation of blink event BOLD responses in the AAN and Thalamic ROI occurred earlier for the Drowsy than the Vigilant state and evinced signal drops in most ROIs, both are emphasized with arrows. In this analysis 15717 vigilant and 7731 drowsy blink epochs were used.

The correlation approaches using canonical HRF and its temporal derivative showed temporal and spatial differences between vigilance states in BOLD-blink interactions (prior sections). Next, we used an event-based approach to measure the average BOLD responses after blinks directly. As shown in **Supplementary Report Part III, Supplementary Figures 19–21** (including the standard error per time point), and Figure 8 below, for AAN, thalamus and cerebellar ROIs, BOLD response initiation for the Vigilant state was close to the blink around 2 to 3s in general, whereas in the Drowsy state, BOLD initiation was before the blink (−2 to −3 s) reaching peak earlier than for the vigilant state. Thus event-based analysis revealed why the correlation analysis with temporal derivative HRF approach above showed a difference: Canonical HRF convolution considers the blink moments as the BOLD onset points (initiation of the neural activity at the blink moment) but the event design reveals that drowsiness delayed the blink motor initiation following the underlying neural activity in AAN and thalamic ROIs. The cerebellum did not show differences in the time of BOLD initiation but the amplitude responses were larger in the Vigilant state for most ROIs whereas the Drowsy state showed marked post-blink dampening of the BOLD activity.

## DISCUSSSION

In this work we used HCP 7T dataset to validate and extend our previous findings related to eye-blink BOLD signal changes within two main vigilance states in healthy human participants. The HCP dataset from the 7T MRI images provided us with a better temporal and spatial resolution that for our prior study based on a 3T MRI. Access to pupillary measures in the HCP allowed us to differentiate the fMRI runs relative to participants’ vigilance state.

We first showed that vigilance affected blink behavior such that blink rate and blink durations were higher while inter-blink intervals were shorter in the Drowsy state compared to the Vigilant state, consistent with the influence of vigilance and arousal surges on blink behavior. As in our prior study of blink related brain activation (Demiral et al., 2023), we showed that peak BOLD signals in AAN, thalamus and cerebellum nuclei occurred in proximity with the blink moment. Additionally by comparing blink-related BOLD surges between vigilant and drowsy states we were able to document differences in the amplitude and temporal shifts as a function of vigilance.

As for our prior findings during pre-blink and blink moments, AAN nuclei, thalamus, visual cortex (core cluster in calcarine sulcus), and cerebellum were the primary regions involved. With the higher spatial resolution from the 7T images we also identified a strong signal in the lateral geniculate body (LGN). In AAN nuclei and in the thalamic nuclei connected to frontal, temporal and motor areas, the blink-related BOLD amplitudes were larger for the Drowsy than the Vigilant state, whereas in the dorsal- and ventral-attention and visual ROIs of the cerebellum as well as the occipital and parietal ROIs of the thalamus BOLD pre-blink amplitudes were larger for the Vigilant state. This points out to the divergent characteristics of the excitability of the cortex, thalamus, AAN and the cerebellum.

Whole brain analyses for the blink related correlations revealed that for the Vigilant state, prominent strong signals emerged in the pre blink and blink moment in ACC, insula, occipital cortex and precuneus (showing as strong correlations) that except for occipital cortex were no longer present post blink. In contrast, for the Drowsy state - positive correlations were apparent in AAN and thalamus and occipital cortex pre-blink with strong anti-correlations emerging in the blink moment in pons, amygdala and parahippocampal regions, and in extensive sensory-motor and temporal cortical regions. The regression analyses using the continuous vigilance measure corroborated that vigilance levels influenced blink-BOLD maximal and minimal correlation values . As we interpret the blink-associated BOLD activation to reflect arousal surges, the significant anticorrelations observed in extensive areas of parietal and temporal cortices for the Drowsy state are consistent with ineffective responses to activate the brain following arousal surges during states of drowsiness. Drowsiness might be associated with lower energy and reduced ATP levels in these cortical regions and thus with a greater demand for oxygen to produce energy. For instance the levels of phosphorylated AMP-activated protein kinase (P-AMPK), which is involved in cellular energy sensing and regulation and of ATP show reciprocal changes such that P-AMPK levels are lower during the sleep-induced ATP surge in the rat brain than during wake or sleep deprivation (Dworak et al., 2010). Thus, during drowsy states P-AMPK might be higher, and homeostatic regulators might try to reduce it, leading to enhanced uptake of oxygen thus affecting the de-oxyhemoglobin – oxyhemoglobin ratios in the tissue, which then influence the magnetic susceptibility effects reflected on the T2* BOLD signal.

The analysis using the blink time series convolved with the temporal derivative of the HRF revealed time-to-peak differences between the Vigilant and the Drowsy state. The time-to peak differences are also evident in the event-based analyses, which showed earlier BOLD initiation in AAN and thalamic nuclei and strong and broad post-blink deactivations for the Drowsy compared to the Vigilant state. Compared to the Vigilant state, arousal-blink relationships were temporally shifted appearing earlier for the Drowsy state such that the blink itself (actual time of the eye closures) appeared as if it emerged a few seconds after the initiation (initial rise) of the BOLD activity, which in the BOLD blink correlation analyses appeared as an early pre-blink correlation. Regression analysis confirmed this finding for most of the AAN, and thalamic ROIs.

This phenomenon (i.e., correlations appearing larger and early for the Drowsy states in the AAN and thalamus regions compared to Vigilant states) might appear counterinitiative at first. However, if we consider the state of drowsiness as a ‘weakened homeostatic state’ where there is a sluggish neural initiation (i.e., executive order) of any mental activity including eye-blinking followed by a sluggish and poor execution of actions (i.e., motor initiation of blinks), this phenomenon could be explained. As mentioned in the introduction, drowsiness can be described as a transitional state between wakefulness and sleep, and micro-sleeps are common in MRI resting state settings (Soon et al., 2021; Tagliazucchi & Laufs, 2014). It is well recognized that drowsiness impairs human performance in a variety of tasks (i.e., psychomotor vigilance task, PVT) (Makeig et al., 2000; Van Dongen et al., 2003). One can assume that the brain gets in a ‘hard-to-operate’ mode during drowsy states. For instance, electrodermal-orienting (EO) response, a measure of attentional shift, was delayed and reduced under drowsiness caused by sleep deprivation (McCarthy & Waters, 1997). In addition, sleep deprivation influenced and delayed the response times and reduced the amplitudes of early and late ERP components (Trujillo et al., 2009). Most importantly, cortical excitability appears to change in drowsy states, as evidenced by larger TMS-evoked potentials (TEP) amplitudes under sleep deprivation (Huber et al., 2013). Sleep pressure in humans as reflected by frequent eye-closures could progressive buildup neuronal excitability in some brain regions; for instance, decreasing levels of neuromodulators during prolonged wakefulness may render cortical neurons hyperpolarized or bistable (i.e., more prone to react to TMS with a high-amplitude synchronous burst of activity (Hill & Tononi, 2005)).

In addition, attentional lapses, as detected during a visuomotor attentional task, are associated with transient increase of excitability (Cardone et al., 2021), indicating that performance decrements and cortical excitability are inter-related. By using TMS together with high-density EEG, Massimini et al. (2005) showed that during quiet wakefulness, an initial response at the stimulation site was followed by a sequence of waves that moved to connected cortical areas several centimeters away, while during non–rapid eye movement sleep (NREM), the initial response was stronger but rapidly extinguished and did not propagate beyond the stimulation site. Thus, aberrant cortical excitability during sleep deprivation can be detrimental to cortical connectivity and motor responses.

The HCP project collected information on participants’ ‘alertness’, as part of the Mini Mental State Examination and the recent sleep profile as part of the Pittsburgh Sleep Quality Index, but they did not collect self-reports of ‘sleepiness’ or ‘alertness’ before or during the scan (https://www.humanconnectome.org/storage/app/media/documentation/s1200/HCP_S1200_Release_Reference_Manual.pdf). Assessment of sleepiness could be quite informative since there are individual differences in responses to sleep deprivation, and concomitantly cortical excitability and reaction times will be affected by this variability (Chia et al., 2021). For instance, information about the amount and quality of sleep in the night prior to the fMRI scan might have been important to assess the influences on the eye-closure durations, drowsiness, and cortical excitability. However, this information is not available and future studies can examine this possibility.

If arousal surges emerge as phasic BOLD increases in AAN and thalamic nuclei during drowsiness, sharp decreases in BOLD post blink would be expected since the brain cannot maintain a well-formed long-lasting homeostatic equilibrium. For example, if the brain initiates and executes ‘blinking action’ as evidenced by a group of neural activity (which would normally coincide with arousal surges and close to the actual blinks in the vigilant state), the consequence of this executive order (as motor execution) becomes weaker and is delayed due to drowsiness. We think that arousal surges aimed at trying to stay awake and keep the eyes open in the MRI environment creates such momentary surges that are weakly temporally coupled with the execution of the blinks. Therefore, the neural activity required to induce arousal-related blink action and following the blink event are qualitatively and quantitatively different in the Drowsy than in the Vigilant state. The longer duration of blinks during the Drowsy state also supports this interpretation.

Drowsiness has been shown to induce changes in fMRI activation and brain connectivity patterns (Liu et al., 2018; Soehner et al., 2019) a finding which was shown to detect vigilance fluctuations online (Chang et al., 2016; Liu & Falahpour, 2020). Here we show that phasic ‘arousal surges’ -theoretically a similar construct to the ‘brief instances of spontaneous brain activity’ (Liu & Duyn, 2013) - to maintain homeostatic balance to stay awake change the brain–blink initiation correspondence. Our results revealed the existence of vigilance-dependent blink timing coincident with fluctuating arousal surges in the brain (in some cases to overcome drowsiness).

The delay in blink initiation after neural activation in the Drowsy state might engage additional dynamic homeostatic mechanisms that might involve thalamus, amygdala and precuneus. While the autonomic nervous system attempts to generate neural signals and activate arousal networks in the drowsy state to maintain consciousness and attention at a reasonable level for basic and sufficient sensory-motor processing, changes in cortical excitability in low-vigilance states delays the processing of neuronal signals interfering with mental tasks. Thus, vigilance is the tonic brain state involving multiple central nervous system in the cortical networks, while arousal surges are the by-products of the ascending arousal nuclei interacting with this high-level cortical states. Blinks might be the behavioral outcome metric of these arousal surges.

## CONCLUSION

In this study, we extended our previous work on brain arousal-blink relationships in AAN and thalamic nuclei and in cerebellum. We also show that during the drowsy state it takes longer to initiate blinks, the blink durations are prolonged, larger BOLD-blink correspondences emerge presumably due to wide and incoherent cortical excitability changes across the cortical, subcortical and cerebral regions, and blinks become more frequent, which might reflect the consequence of an exaggerated effort to stay awake in the MRI scanner. Timing of blinks and blink-related BOLD signatures in this context play a valuable role in understanding arousal networks in health and disease states.

## Figures and Tables

**Table 1:** Significant Clusters at 0s-lag

VIGILANT					
Area	k (ml)	X	Y	Z	ABS MEAN
**Visual**	80643	1.3	−74.3	6.9	18.1
**ACC**	7519	0.1	19.5	36.7	12.2
**Precuneus; Paracentral Lobule**	3740	2.4	−48.3	56.1	11.1
**L BA 9/10**	3631	−34.9	42.3	27.4	11.2
**L Anterior Insula**	2138	−37.5	14.7	4.7	11.0
**Cerebellum - L Lobule VI**	2005	−36.8	−51.5	−31.4	11.0
**Cerebellum - R Lobule VI**	1833	39.4	−51.2	−31.8	11.4
**R BA 9/10**	1668	32.5	42.2	28	10.9
**R Anterior Insula**	1434	39.1	15.1	5.2	10.9
**R Lateral Geniculum Body**	1098	24.1	−25	−5.3	16.4
**L Lateral Geniculum Body**	936	−23.3	−25.2	−6.8	15.8
**L IPL**	738	−56.1	−36.8	35.8	10.6
R Superior Temporal Gyrus	617	42.3	18.5	−34.6	10.8
**L Cingulate Gyrus**	593	−11.7	−36.7	45	11.0
**L Superior Frontal Gyrus**	492	−17.7	2	68.8	10.9
**R Cerebral Tonsil**	325	36.1	−47.5	−51.6	11.0
L Middle Temporal Gyrus	316	−65.1	−13.5	−20.8	10.7
R Middle Temporal Gyrus	172	44.3	−66.6	3.1	10.7
L Middle Temporal Gyrus	127	−59.3	−0.6	−26.8	10.3
**Brain Stem; Rostral ventromedial medulla**	125	4	−38.7	−43.1	10.6
R Amygdala	113	24	−11.8	−17.9	10.2
R Middle Frontal Gyrus	80	40.2	37.8	−16.1	10.3
L Superior Frontal Gyrus	73	−11.6	48.7	41.1	10.1
R Anterior Fusiform Gyrus	43	52.4	−0.7	−31	10.2
**R Superior Frontal Gyrus**	33	20.1	2	67.6	10.2
R Parahippocampal Gyrus	28	16.3	−10.1	−18.9	10.2
L Inferior Frontal Gyrus	21	−47.7	37.9	−15	10.2
R Frontal Superior Gyrus	20	12.7	48.8	44.5	10.0
R Middle Frontal Gyrus	17	47.9	42.7	−16.9	10.1
**R Frontal Superior Gyrus**	14	26.8	3.7	70.5	10.3
**R Uvula - Cerebellum**	12	6	−71.5	−41.5	10.2
R. Middle Frontal Gyrus	12	51	40.5	−14.5	10.1
DROWSY					
Area	k (ml)	X	Y	Z	ABS MEAN
L and R Pre-Central and Superior Temporal Cortex	98627	2.6	−22.1	33.5	11.6
R Posterior Fusiform Gyrus / Ventral Temporal Lobe	16435	40.9	−59.3	−8	11.2
L Parahippocampal Gyrus	5395	−31.7	−54.9	−13.3	10.8
L Middle Occipital Gyrus	5166	−45.3	−74.4	0.8	11.0
R Amygdala and Parahippocampal Gyrus	2175	24.4	−11.7	−19.9	11.4
L Amygdala and Parahippocampal Gyrus	1660	−22.4	−11	−20.3	11.1
**L and R Ventral Anterior Thalamus**	1519	1	−6.6	10.8	10.8
**R Medial Dorsal Thalamus**	621	0.9	−19.1	12.3	10.5
**Lateral Ventricles**	276	−2.9	6.9	19	10.4
**Cerebellum L Culmen (VI)**	193	−36.5	−56.1	−30.4	10.4
**R Primary Visual Cortex / Calcarine Gyrus**	188	12.5	−82.2	7.2	10.3
**R Lateral Geniculum Body**	165	25.3	−24.1	−5.8	11.5
**L Lateral Geniculum Body**	100	−24.2	−24.6	−6.5	11.1
**Medulla; Ganglionic Nucleus**	59	0.2	−45.1	−50.8	10.1
R Parahippocampal Gyrus	48	26.3	3.4	−19.6	10.2
L Middle Occipital Gyrus	44	−28.7	−84.4	15.9	10.2
R Thalamus Posterior Medial	37	13.8	−22.6	0.6	10.3
**L Primary Visual / Calcarine**	14	−7.7	−82.1	4.5	10.0
L Thalamus Posterior Medial	12	−12.5	−23.5	−1	10.2
R Inferior Frontal Gyrus	11	28.5	8.8	−24.3	10.1

Abbreviations: k: cluster size, X, Y, Z are MNI coordinates of the center of the activity cluster, ABS MEAN: absolute mean t-value, SEM: standard error of the mean, Max Int: Maximum intensity value, Region: Anatomical labels of the significant clusters. Clusters are ordered with respect to their cluster size. Labels of the positive clusters are shown in bold font those for negative clusters in regular font.
